# Identification and characterization of interferon-γ signaling-based personalized heterogeneity and therapeutic strategies in patients with pancreatic cancer

**DOI:** 10.3389/fonc.2023.1227606

**Published:** 2023-10-24

**Authors:** Xu Chen, Qihang Yuan, Hewen Guan, Xueying Shi, Jiaao Sun, Zhiqiang Wu, Jie Ren, Shilin Xia, Dong Shang

**Affiliations:** ^1^ Department of General Surgery, First Affiliated Hospital of Dalian Medical University, Dalian, Liaoning, China; ^2^ Laboratory of Integrative Medicine, First Affiliated Hospital of Dalian Medical University, Dalian, Liaoning, China; ^3^ Department of Dermatology, First Affiliated Hospital of Dalian Medical University, Dalian, Liaoning, China; ^4^ Department of Urology, The First Affiliated Hospital of Dalian Medical University, Dalian, Liaoning, China; ^5^ Department of Oncology, First Affiliated Hospital of Dalian Medical University, Dalian, Liaoning, China

**Keywords:** pancreatic cancer, pan-cancer, interferon-γ (IFN-γ) signaling pathway, IFN-γ-related genes, prediction model

## Abstract

**Background:**

Interferon-γ (IFN-γ) is a key cytokine with diverse biological functions, including antiviral defense, antitumor activity, immune regulation, and modulation of cellular processes. Nonetheless, its role in pancreatic cancer (PC) therapy remains debated. Therefore, it is worthwhile to explore the role of Interferon-γ related genes (IFN-γGs) in the progression of PC development.

**Methodology:**

Transcriptomic data from 930 PC were sourced from TCGA, GEO, ICGC, and ArrayExpress, and 93 IFN-γGs were obtained from the MSigDB. We researched the characteristics of IFN-γGs in pan-cancer. Subsequently, the cohort of 930 PC was stratified into two distinct subgroups using the NMF algorithm. We then examined disparities in the activation of cancer-associated pathways within these subpopulations through GSVA analysis. We scrutinized immune infiltration in both subsets and probed classical molecular target drug sensitivity variations. Finally, we devised and validated a novel IFN-γ related prediction model using LASSO and Cox regression analyses. Furthermore, we conducted RT-qPCR and immunohistochemistry assays to validate the expression of seven target genes included in the prediction model.

**Results:**

We demonstrated the CNV, SNV, methylation, expression levels, and prognostic characteristics of IFN-γGs in pan-cancers. Notably, Cluster 2 demonstrated superior prognostic outcomes and heightened immune cell infiltration compared to Clusters 1. We also assessed the IC50 values of classical molecular targeted drugs to establish links between IFN-γGs expression levels and drug responsiveness. Additionally, by applying our prediction model, we segregated PC patients into high-risk and low-risk groups, identifying potential benefits of cisplatin, docetaxel, pazopanib, midostaurin, epothilone.B, thapsigargin, bryostatin.1, and AICAR for high-risk PC patients, and metformin, roscovitine, salubrinal, and cyclopamine for those in the low-risk group. The expression levels of these model genes were further verified through HPA website data and qRT-PCR assays in PC cell lines and tissues.

**Conclusion:**

This study unveils IFN-γGs related molecular subsets in pancreatic cancer for the first time, shedding light on the pivotal role of IFN-γGs in the progression of PC. Furthermore, we establish an IFN-γGs related prognostic model for predicting the survival of PC, offering a theoretical foundation for exploring the precise mechanisms of IFN-γGs in PC.

## 1 Introduction

PC is a common kinds of gastrointestinal tumors that accounts for a significant portion of tumor-related fatalities worldwide. Surgical resection remains the primary treatment for PC (1). Unfortunately, the majority of patients are unable to undergo surgery due to late diagnosis. Immunotherapy has been established as an effective treatment for several malignancies; however, it only benefits a small subset of PC patients (2). Moreover, the inconsistent success in molecular targeted therapy can be attributed to the heterogeneity of tumors (3). Therefore, it is important to explore and authenticate new prognostic indicators to enhance the prognosis of clinical consequences and chemotherapy responsiveness among PC patients.

Interferons, classified as vital proteins within the cytokine family, serve diverse biological functions, with a particular emphasis on their pivotal role in the immune system. There are three primary types of interferons, namely types I, II, and III, each with distinct roles in tumorigenesis. Extensive research has demonstrated that type I and type III interferons unequivocally exhibit the ability to impede tumor growth (4, 5). In the case of type II interferon, known as IFN-γ, its impact on tumor immune evasion and bidirectional immune surveillance has generated controversy surrounding its antitumor properties (6–8). Therefore, this study is dedicated to exploring the potential role of IFN-γ in PC. And it primarily exerts its biological effects through cytostatic/cytotoxic and antitumor processes within the adaptive immune response, which is mediated by cells (9). The activation of the Janus kinase 1 (JAK1) pathway and the signal transducer and activator of transcription 1 (STAT1) is the primary mechanism through which IFN-γ signals (10). IFN-γ signaling is integral to various biological processes, including inflammation regulation, innate and acquired immunity, cell cycle control, apoptosis, and defense against viral and bacterial infections (9). Studies have reported that IFN-γ can inhibit tumor by enhancing the efficacy of anti-pancreatic cancer targeted drugs (11, 12). Although IFN-γ signaling has long been considered crucial to antitumor immunity, recent evidence suggests that it has a dual role in promoting cancer development and immune evasion (13). A study has reported that IFN-γ could promote PC epithelial-mesenchymal transition (14). Hence, owing to the ambiguity surrounding the function of IFN-γ in PC, it is imperative to explore the IFN-γ signaling pathway’s role in this context. In light of the pivotal role played by the IFN-γ signaling pathway in tumor immunity and the treatment resistance arising from its impairment in tumor cells, there is substantial value and significance in investigating the involvement of IFN-γ in the progression of PC and in establishing pertinent predictive models.

Based on the previously mentioned information, we were able to classify 930 patients with PC into two separate groups relying on the expression levels of IFN-γGs. We then evaluated the relationship of these subgroups with patient prognosis, differential gene expression, HALLMARKER signaling pathways, immune microenvironments, and drug sensitivities. Next, using LASSO-COX analysis, we identified seven hub genes, namely EREG, IAPP, KRT17, ANXA1, C7, and ALB, to establish and verify a novel predictive model for PC. We demonstrated the stability and reliability of this predictive model through both internally and externally validating the prediction model in PC. By utilizing the model, we categorized patients into groups of low and high risk, and analyzed their associations with prognosis, immune microenvironments, as well as drug sensitivities. Finally, the study confirmed the levels of expression for seven hub genes in pancreatic cancer cell lines and clinical samples. Collectively, our study established a novel prognostic signature for PC, and the outcomes may provide new avenues for clinical decision-making and prognostic evaluation in the context of the malignancy.

## 2 Materials and methods

### 2.1 Data aggregation and processing

The publicly available data on gene expression as well as clinical information sourced from multiple databases were gathered, such as TCGA, GEO, ICGC, ArrayExpress, and GTEx. To ensure the accuracy of our analysis, we eliminated patients lacking survival data and addressed batch effects by employing the ComBat technique from the “SVA” package (15, 16). Multiple datasets were combined, including GSE57495, GSE28735, GSE62452, MTAB-6134, and TCGA-PC, which collectively comprised 635 PC samples. We randomly assigned 319 samples to the training cohort, 316 samples to the test1 cohort, and utilized all 635 samples as the test2 cohort. External validation was performed using ICGC-CA and ICGC-AU datasets, which provided 295 samples for the test3 cohort. Overall, our study included 930 PC samples with clinical information. Lastly, we extracted 93 IFN-γGs from the Molecular Signature Database (MSigDB).

### 2.2 Comprehensive analysis of IFN-γGs in multiple human cancers

We comprehensively summarized IFN-γGs by utilizing a similar approach to prior studies through the downloading and organization of multi-omics data from the TCGA pan-cancer cohort. Specifically, we conducted a detailed analysis of interferon gene copy number variation (CNV), single nucleotide variation (SNV), and changes in methylation patterns at genomic level. At the transcriptomic level, we extensively investigated the expression, prognosis of the IFN-γGs. It is noteworthy that we compared tumor tissues and adjacent normal tissues from the TCGA cohort to generate the pan-cancer expression profile of IFN-γGs. The results supply valuable comprehension for the regulatory mechanisms and prospective clinical significance of IFN-γGs in the context of pan-cancer biology.

### 2.3 Non-negative matrix factorization clustering determination of IFN-γGs modification subtypes

Our goal was to investigate the association of IFN-γGs expression with clinical characteristics in PC (17). We used the NMF algorithm to partition the 930 samples of PC into distinct clusters. NMF algorithm aims to identify potential genes expression models by decomposing the original matrix into two nonnegative matrices. The specific parameters of the NMF clustering algorithm are as follows: rank=2:10, method=“brunet”, nrun=100. We choose values of k at the point where the cophenetic correlation coefficient starts to decrease in magnitude (18). Next, we utilized the “survival” package in R to complete the Kaplan-Meier (K-M) survival analysis. Survival analysis contributes to a better understanding of the significant value of NMF clustering in the clinical outcome of PC.

### 2.4 Gene set variation analysis

In order to evaluate the IFN-γ pathway activation among PC patients, we employed the “GSVA” package in R to calculate individual IFN-γ pathway scores (19). These scores served as an effective indicator of pathway activity and enabled us to contrast the differences in IFN-γ scores among the two different groupings of patients using R’s “wilcox.test” function. In addition, we used the “GSVA” software to compute the enrichment scores for 50 hallmark pathways and applied a similar methodology to identify any potential signaling pathway discrepancies between clusters (20). We further investigated the expression of IFN-γGs in the two clusters to gain deeper insights into the underlying mechanisms involved.

### 2.5 Analyzing tumor immune microenvironments between the C1 and C2

The tumor microenvironment (TME) is predominantly comprised of immune cells, stromal cells, and tumor cells. To investigate the immunological features among distinct clustering subtypes from a holistic standpoint, we have introduced the well-established Estimate algorithm. This algorithm is executed through the “estimate” package in the R programming language (17), enabling the quantification of ImmuneScore, StromalScore, EstimateScore, and tumor purity using gene expression profiles as the foundation. To visually illustrate the scores associated with the TME in a comprehensible manner, we utilized the ggpubr package to craft refined violin plots. In order to comprehensively evaluate immune cell infiltration (ICI), we utilized several immune-related algorithms, including TIMER, CIBERSORT, CIBERSORT-ABS, QUANTISEQ, MCPCOUNTER, XCELL, and EPIC algorithms (21, 22). Furthermore, tumor cells can employ immune checkpoints (ICs) to evade attacks from the immune system, aiding their escape from immune surveillance and survival. Therefore, we also compared the differences in ICs expression levels between different subtypes in PC.

### 2.6 Drug sensitivity and differentially expressed genes analysis

Utilizing the R package “pRRophetic” to forecast drug sensitivity and enhance our comprehension of the connection between tumor drug treatment and the expression of IFN-γGs (23). This package is based on the Cancer Genome Project (CGP) and includes 138 anticancer drugs that were tested against 727 cell lines. The semimaximum inhibitory concentration (IC50) of the samples was calculated using the ridge regression method (24). A lower semi-inhibitory mass concentration of the drug in cancer cells generally implies a smaller IC50, indicating that the cancer cells are more responsive to the medicine. Additionally, we employed the “limma” program to identify the DEGs by applying filtering criteria of fold-change (FC) > 1.5 and false discovery rate (FDR) < 0.05 between the C1 and C2 subtypes (25).

### 2.7 Establishing and validating a novel IFN-γ-based risk signature

In order to address collinearity, over-fitting, and determine the optimal variables from the aforementioned DEGs, we employed LASSO regression analysis in the training cohort (26). Next, utilizing the multivariate Cox regression analysis, we developed a model to determine the risk score for each sample and calculated the risk score for each sample utilizing the predict function in R (27). After grouping the patients in the training cohort relying the median risk score, they were separated into high- and low-risk categories. This same process was used for patients in the test1, test2, and test3 cohorts, where they were divided into high- and low-risk categories using the median risk scores derived from the training cohort. These categories were then utilized for further analysis.

To validate the model both internally and externally, we carried out similar analyses on different cohorts, including the training, test1, test2, and test3 sets: (1) we generated a heatmap by the R package ‘pheatmap’ to illustrate he levels of gene expression in the model; (2) we performed survival analysis using the KM approach; and (3) To evaluate our model’s diagnostic performance, we generated receiver-operating characteristic (ROC) curves, which were used to compute the area under the curve (AUC) (28).

### 2.8 Analyzing the immune microenvironment of tumors and drug sensitivity between patients with low and high risk

Studying the diversity in the immune microenvironment among high-risk and low-risk groups, we conducted a study of ImmuneScore, StromalScore, EstimateScore, and tumor purity. In addition, various algorithms were utilized to investigate the immunological differences among high-risk and low-risk groups (29). The study also investigated ICG expression in different risk populations, as previously mentioned. Lastly, we conducted drug sensitivity analysis to pinpoint potential effective medicines for both high-risk and low-risk populations. We just deemed substances that displayed statistical significance across the training, test1, test2, and test3 groups as dependable and authentic targeted agents.

### 2.9 Validation of seven model genes by qRT-PCR and HPA platform

The prognostic significances of seven model genes in 930 PC samples using KM and univariate Cox regression analysis were investigated. We proceeded to generate heatmaps of the model genes in the TCGA, GSE28735, and GSE62452 datasets to investigate their differential expression in tumor and normal tissues. Following this, at the transcriptional level, we intend to confirm the conclusion of different expression levels of the seven model IFN-γGs in seven paired clinical samples and four distinct types of pancreatic cancer cell lines utilizing the Quantitative real-time PCR(qRT-PCR).

The normal human pancreatic ductal cell line called HPDE6-C7 was provided by the BeNa Culture Collection (BNCC). Procell Life Science & Technology Co., Ltd supplied four distinct human pancreatic cancer cell lines, specifically Bxpc-3, PANC-1, CFPAC-1, and Mia-Paca-2. HPDE6-C7, PANC-1, and Mia-Paca-2 were cultivated by DMEM supplemented with 10% fetal bovine serum (FBS), as per BNCC’s product specifications. CF-PAC1 and Bxpc-3 cell lines were cultured in IMDM and 1640, respectively, with 10% FBS. The cell lines were kept at a temperature of 37°C under an atmosphere containing 5% CO2.

Between January and December 2022, we randomly selected seven fresh pancreatic tumor tissues and their corresponding paracancerous tissues, which were immediately treated with liquid nitrogen freezing after surgical excision. All samples were obtained from the First Affiliated Hospital of Dalian Medical University. In our research, all patients received standard preoperative care, and patients with PC did not undergo chemotherapy or radiation therapy. This research received support and informed consent from the Ethics Committee at the First Affiliated Hospital of Dalian Medical University.

According to the manufacturer’s instructions, TRIzol reagent (ADAMAS LIFE) was used to extract total RNA from four kinds of pancreatic cancer cell lines and the HPDE6-C7. The total RNA from the seven paired clinical samples was also extracted using the TRIzol method with liquid nitrogen grinding. The cDNA was synthesized by reverse transcription of all RNA samples using the All-in-One First-Strand Synthesis Master Mix (with dsDNase) (Yugong Life Technology Co., Ltd). The mRNA expression levels of the seven genes were quantified using SYBR™ Green (Iscience Biotech) on the Bioer 9600 FQD-96A fluorescence quantitative PCR instrument. An internal control, β-actin was utilized. The primer sequences (Sangon Biotech) for β-actin and the seven hub genes were as follows: for human β-actin, 5’-CCTGGGCATGGAGTCCTGTG-3’, 5’-TCTTCATTGTGCTGGGTGCC-3’; for human ALB, 5’-AGGCAACAAAAGAGCAACTGAAAGC-3’, 5’-CGGCAAAGCAGGTCTCCTTATCG-3’; for human IAPP, 5’-GCAACAACTTTGGTGCCATTCTCTC-3’, 5’-GGGCAAGTAATTCAGTGGCTCTCTC-3’; for human C7, 5’-TCAAGTGCCTCCTCTCCAGTCAAC-3’, 5’-ACCGCCTGCGAGTCTGAGTC-3’ (Reverse); for human ANXA1, 5’-CTCGGATGTCGCTGCCTTGC-3’, 5’-CTGCTTTGATCTGTTGACGCTGTG-3’; for human EREG, 5’-GTGGGTTATACTGGTGTCCGATGTG-3’, 5’-ATGTGGAACCGACGACTGTGATAAG-3’; for human ADM, 5’-TGGGTTCGCTCGCCTTCCTAG-3’, 5’-ACATCCGCAGTTCCCTCTTCCC-3’.

To verify the expression levels of the seven genes at the protein level, we intended to utilize The Human Protein Atlas (HPA: https://www.proteinatlas.org/). Nonetheless, we were only able to obtain protein expression data for six genes, as we could not find EREG’s protein expression data on the HPA database. Additionally, the HPA platform uses both the intensity of staining and the proportion of stained cells to categorize antibody staining in diverse kinds of human tissue cancer as unobserved, low, medium, or high. Our study included the cellular localizations of the hub genes, excluding ADM and C7, as revealed by immunofluorescence using HPA data.

## 3 Results

### 3.1 Pan-cancer overview of the IFN-γ-related genes

**Figure 1:**
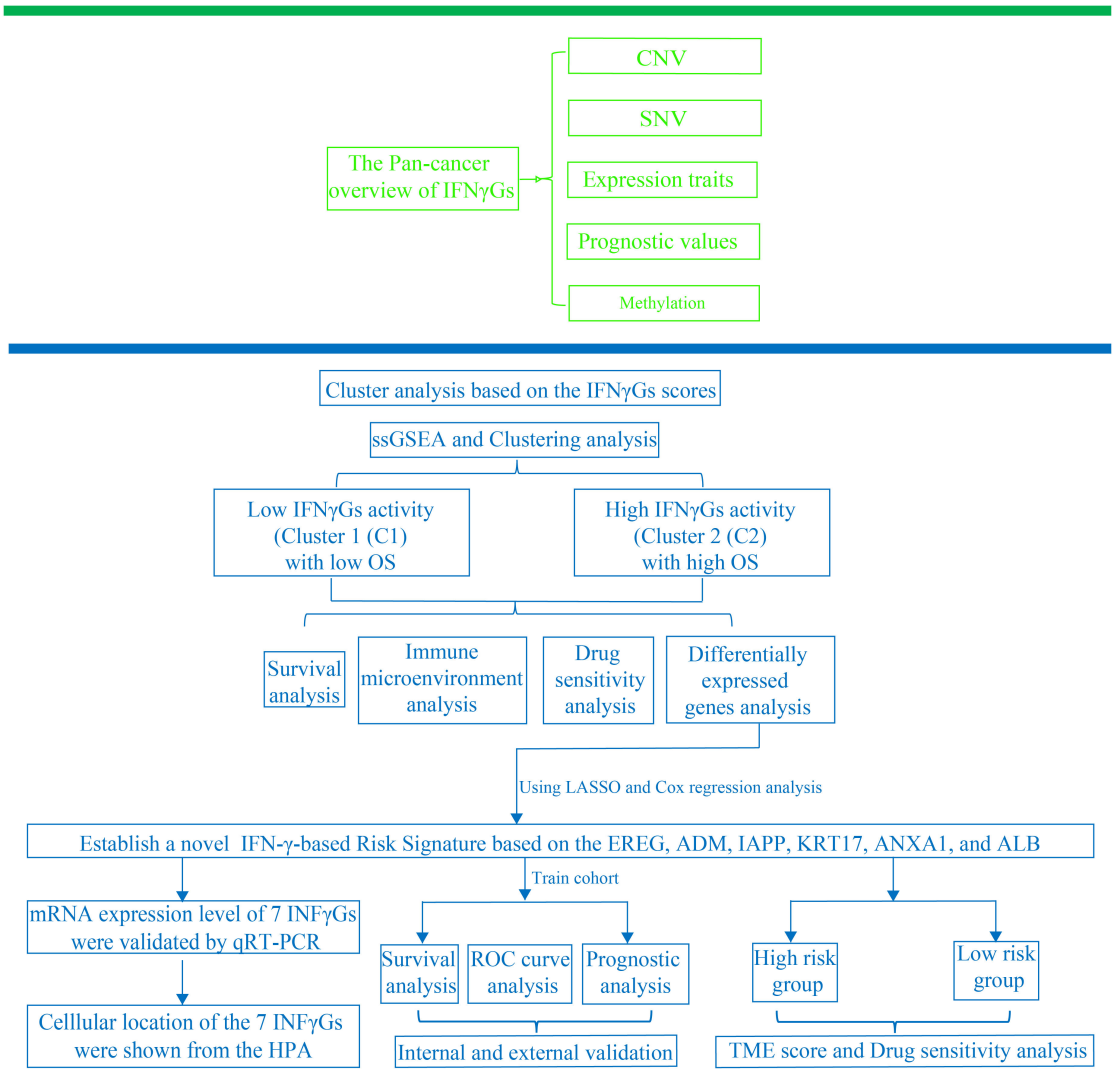
The research’s analytical workflow in detail.

**Figure 2:**
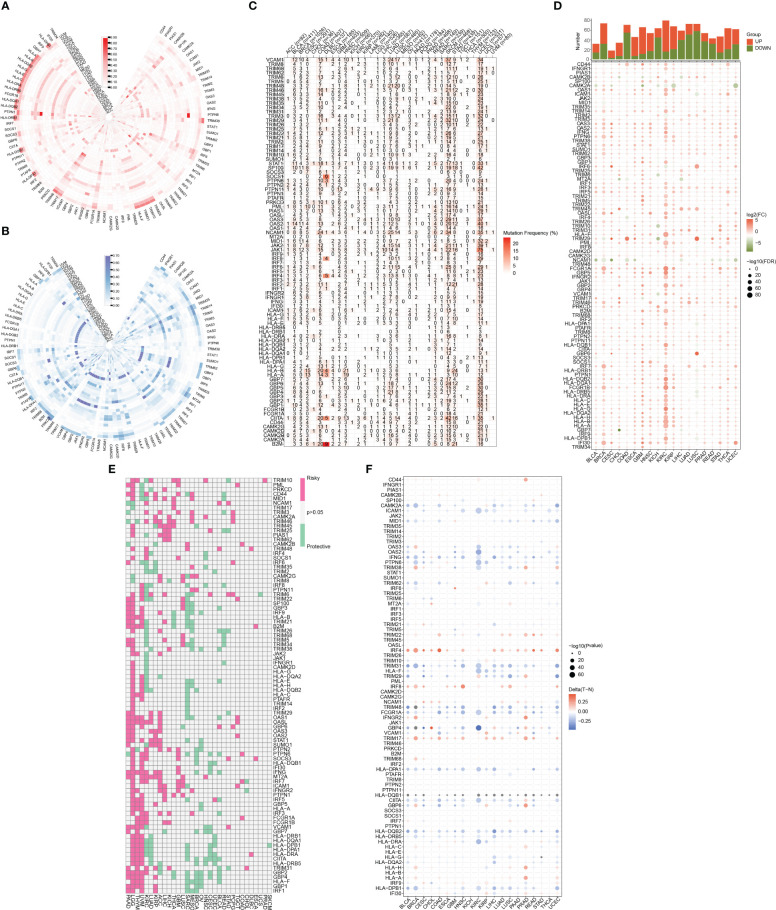
A summary of IFN-γ-related genes across various types of cancer. **(A)** Frequency data in pan-cancer for CNV gain. The gain frequency of genes related to IFN-γ is represented by the color red. **(B)** Frequency data in pan-cancer for CNV loss. The loss frequency of genes related to IFN-γ is represented by the color blue. **(C)** Frequency data in pan-cancer for SNV. **(D)** Expression levels of IFN-γ-related genes in tumors and adjacent normal tissues in various cancer types(P<0.05). **(E)** Analysis of IFN-γ-related genes survival rates across all cancer types. Genes with a P-value > 0.05 are represented in white, while pink and green denote high-risk and protective genes, separately. **(F)** IFN-γ-related gene methylation levels vary across different tumors and the red-to-blue gradient represents high-to-low methylation levels, with red indicating high methylation and blue indicating low methylation.

**Figure 1** illustrates the flow chart for the study, wherein we utilized genomics, transcriptomics, and clinical data from public databases to analyze and reveal the molecular signature and critical role of 93 IFN-γGs in diverse human tumors. Initially, we evaluated the mutational characteristics of these genes in human cancers by evaluating the proportion of CNV (**Figures 2A, B**). Our analysis showed that IFN-γGs have a relatively higher frequency of gain mutations in several cancers, including SKCM, OV, LIHC, KIRC, KICH, and ACC, with UVM and UCS exhibiting the highest frequencies. Notably, PTPN1, TRIM46, TRIM17, FCGR1A, IRF5, IRF6, and CAMK2B had a higher frequency of gain mutations in most human cancer types. In contrast, IFN-γGs have a higher frequency of loss mutations in UVM, UCS, PCPG, OV, LGG, SKCM, and SARC, with KICH showing the highest frequency. Moreover, IRF2, IRF8, PRKCD, IFNGR1, TRIM35, TRIM2, TRIM3, TRIM29, and CAMK2D had a higher frequency of loss mutations in most human cancer types. The molecular signature of IFN-γGs for SNV showed a higher frequency of SNVs in COAD, LUAD, SKCM, STAD, and UCEC, with VCAM1, TRIM48, STAT1, PTPN2, JAK2, JAK1, CIITA, and TRIM46 having significantly higher frequencies than other IFN-γGs (**Figure 2C**). Further, we examined the expression characteristics of 93 IFN-γGs in samples from cancerous tissue and nearby healthy tissue in various types of cancer. The mRNA expression levels of most IFN-γGs were significantly increased in almost all tumor tissues, especially in BRCA and KIRC. In contrast, the mRNA expression level of NCAM1 decreased in vast majority of human tumors as compared to their respective normal samples, such as BRCA, BLCA, COAD, UCEC and so on (**Figure 2D**). Additionally, almost all IFN-γGs showed differential expression in cancer and para-cancer, which could significantly influence the development and prognosis of pan-cancer. We studied the prognostic capabilities of IFN-γGs in human tumors using univariate cox regression analysis and identified risky and protective IFN-γGs (**Figure 2E**). We found the IFN-γGs played the different roles in pan-cancers. For instance, the PTPN6 was acted as the protective factor for PC, SARC, BRCA, CESC and BLCA, while acted as the risk factor for LGG, UVM, KIRC, GBM and COAD. We also explored the methylation patterns of IFN-γGs in the 20 pan-cancer, and our analysis showed that most IFN-γGs displayed hypomethylation in almost all cancer types, including PC (**Figure 2F**). Notably, IRF4, IRF8, TRIM17 exhibited hypermethylation in all human tumors.

### 3.2 NMF clustering identification of molecular typing according to the IFN-γGs

**Figure 3:**
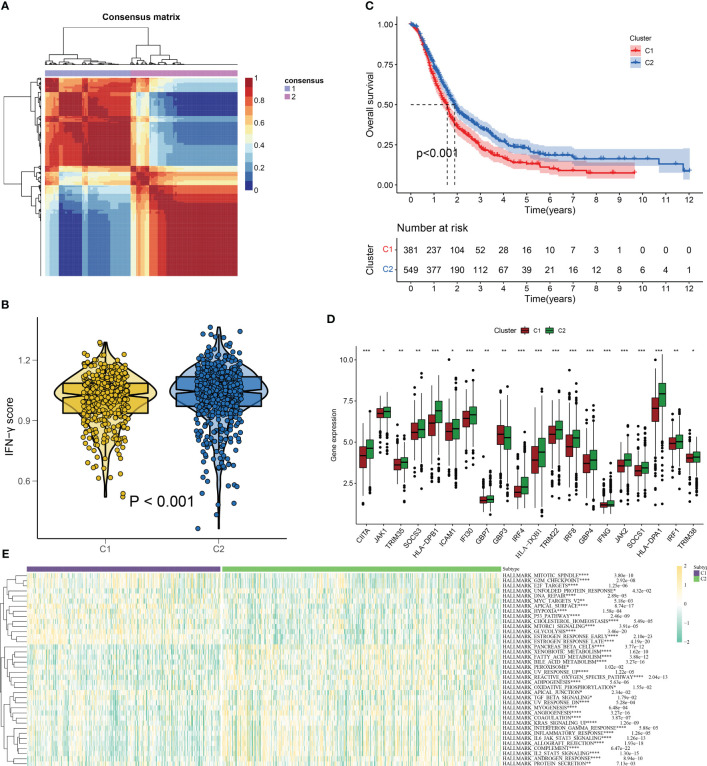
IFN-γ scores-based clustering analysis. **(A)** 930 PC patients are divided into two subgroups(C1 and C2) using the NMF algorithm. **(B)** A violin plot from the “ggpubr” package displays enrichment scores for two clusters (C1 and C2), with low to high scores on the y-axis. **(C)** Survival curve comparison of patients in clusters 1 and 2(C1 represented by the red line and C2 represented by the blue line. The abscissa signifies the number of years, while the ordinate denotes the survival rate. It is evident from the curve that C2 has a better survival rate compared to C1). **(D)** Differentially expressed IFN-γGs in the C1 and C2 subgroups(C1 represented by the red and C2 represented by the green). **(E)** The classical cancer-related pathways between C1 and C2(C1 represented by the blue and C2 represented by the green). (*: p-value < 0.05; **: p-value < 0.01; ***: p-value < 0.001; ****: p-value < 0.0001).

We employed the NMF algorithm to divide the 930 PC patients into two subgroups based on cophenetic, dispersion, and silhouette indicators (**Figures 3A**, **S1**). Our findings from the GSVA and KM analyses suggested that patients in subgroup C2 exhibited higher IFN-γ scores and better overall survival (OS), showing the presence of IFN-γGs might suggest a protective role for PC patients (**Figure 3B, C**). Additionally, **Figure 3D** highlights the differentially expressed IFN-γGs in the two subgroups. Furthermore, the ssGSEA results based on pathways showed that the activation of pathways were higher in the C2 subtype than that in C1 subtype, including PANCREAS_BETA_CELLS, ANGIOGENESIS, INFLAMMATORY_RESPONSE, KRAS_SIGNALING, REACTIVE_OXYGEN, TGF_BETA_SIGNALING, and PEROXISOME, emphasizing the IFN-γ was closely linked with these common pathways (**Figure 3E**).

### 3.3 Analyzing tumor immune microenvironments between the C1 and C2 subgroups

**Figure 4:**
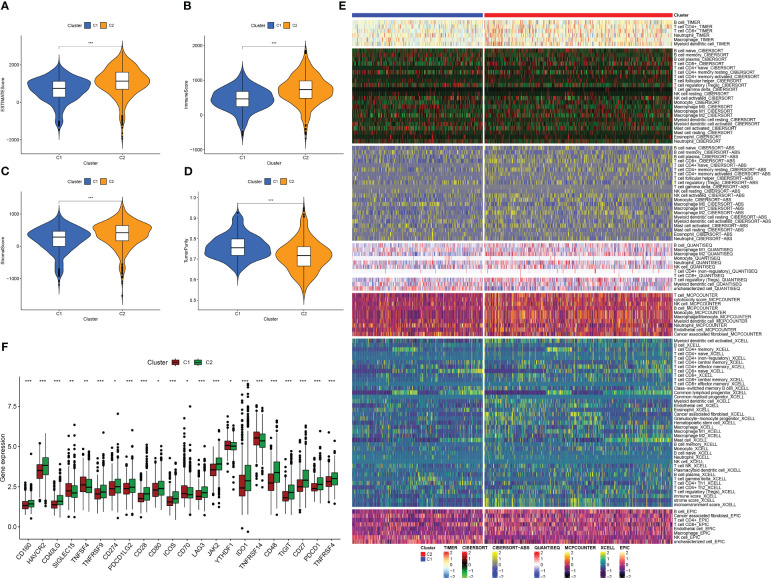
Analysis of tumor immune microenvironment in two IFN-γ-related clusters. **(A–D)** Boxplots showing EstimateScore, ImmuneScore, StromalScore, and tumor purity in C1 and C2. **(E)** Comparison of immune cell infiltration percentages in C1 and C2 using TIMER, CIBERSORT, CIBERSORT-ABS, XCELL, EPIC, and MCPCOUNTER algorithms. **(F)** Comparison of immune checkpoint genes expression in C1 and C2. (*: p-value < 0.05; **: p-value < 0.01; ***: p-value < 0.001).

We used the “ESTIMATE” R software package to evaluate the immune properties of C1 and C2 subtypes utilizing transcriptome data. The outcomes we achieved demonstrated that the C2 subtype displayed elevated levels of ImmuneScore, StromalScore, and ESTIMATEScore, while tumor purity was reduced (**Figures 4A–D**). In order to examine immune cell presence within the TME, we employed diverse algorithms to determine the proportion of immune cells infiltrating in both subcategories. As shown in **Figure 4E**, we detected variations in the infiltration levels of immune cells within the two subgroups, C1 and C2. Notably, within the C2 subgroup, there was a greater prevalence of immune cell infiltration, encompassing the CD4+ and CD8+ T cells, B cells, as well as macrophages/monocytes, in comparison to the C1 subgroup on the whole. Furthermore, the C2 subtype demonstrated increased expression of ICs (**Figure 4F**), which are vital in regulating immune function. Our discoveries suggest that targeting ICs might be prospective approach to improve the prognosis of patients with PC. Additionally, we identified CD27, CD40LG, CD48, and JAK2 as potential prognostic markers associated with better outcomes, while PDCD1LG2 was associated with worse outcomes (**Figure S2**). In summary, the research outcomes provide valuable insight into the immune features of C1 and C2 subcategories and offer potential therapeutic targets for improving the prognosis of patients with PC.

### 3.4 Drug sensitivity analysis

**Figure 5:**
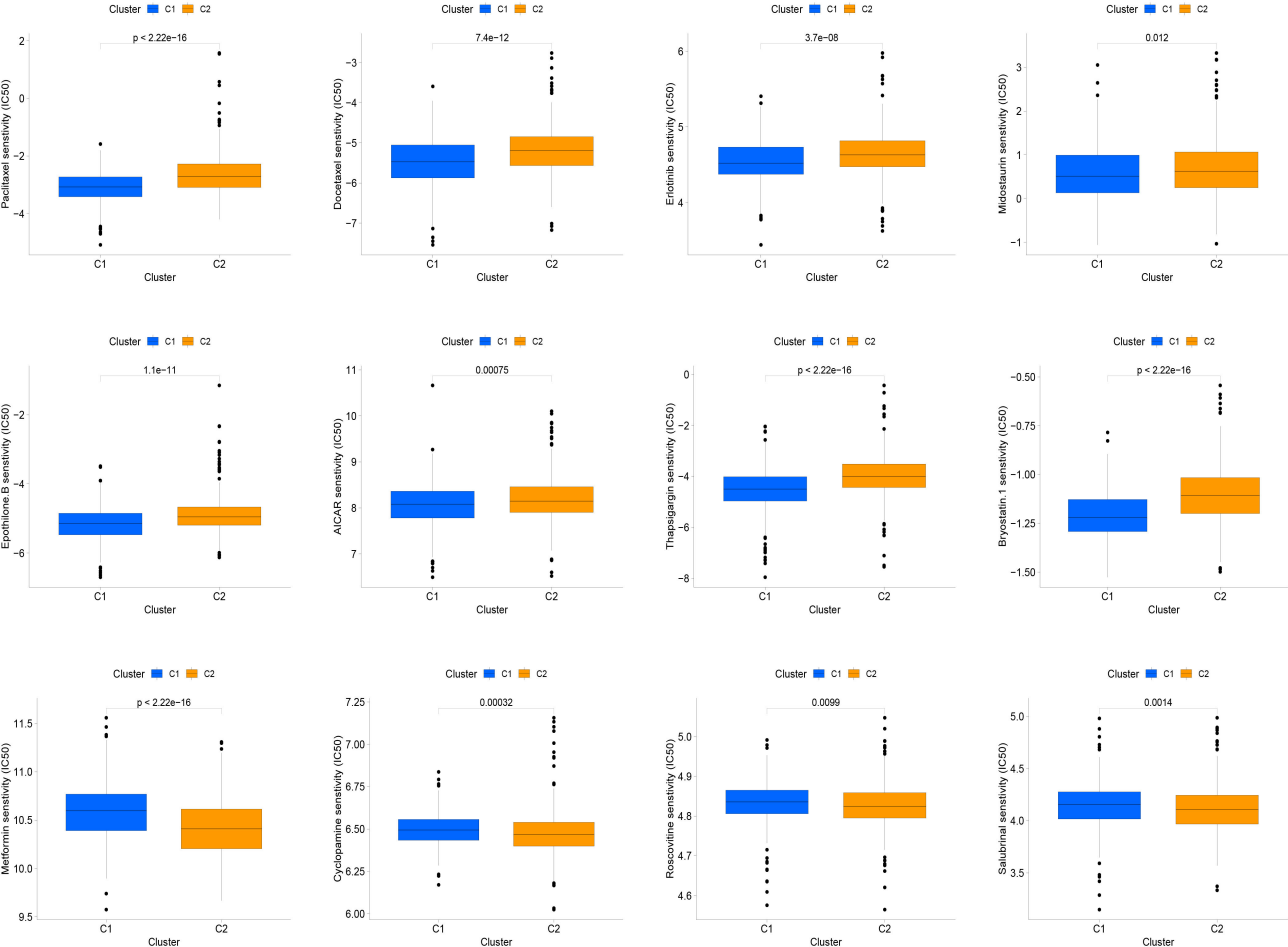
The relationship between IFN-γ clusters and responsiveness to drugs. Box plots of estimated IC50 values for 12 commonly used chemotherapeutic drugs have been generated for C1 (blue) and C2 (yellow). The 12 types of chemotherapeutic agents can be listed as follows: Paclitaxel, Docetaxel, Erlotinib, Midostaurin, Epothilone.B, AICAR, Thapsigargin, and Bryostatin.1, Metformin, Cyclopamine, Roscovitine, and Salubrinal.

Molecularly targeted therapies were a popular choice for PC treatment, we assessed the response of two subtypes to chemotherapy using the “pRRophetic” package. The medications evaluated included targeted therapies specific to PC and conventional drugs used in oncology research. These medications included docetaxel, erlotinib, paclitaxel, metformin, bryostatin.1, thapsigargin, roscovitine, salubrinal, midostaurin, epothilone.B, cyclopamine and AICAR. Our findings suggested that paclitaxel, docetaxel, erlotinib, midostaurin, epothilone.B, AICAR, thapsigargin, and bryostatin.1 might be beneficial for the C1 subtype, while the C2 subtype was found to be more responsive to metformin, cyclopamine, roscovitine, and salubrinal, as shown in the **Figure 5**.

### 3.5 DEGs analysis and risk model establishment

**Figure 6:**
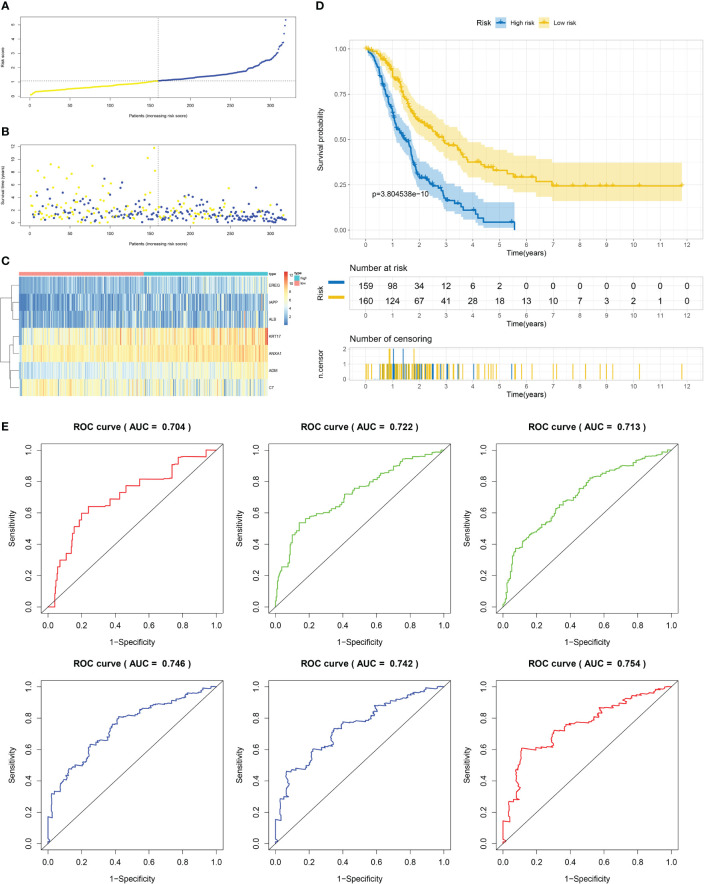
Establishment of risk model development of PC based on IFN-γGs in the train cohort. **(A)** Patients distribution among two risk groups according to appropriate score cut-off values. **(B)** Distribution of survival status and risk scores among patients. (Higher risk scores were associated with increased of mortality). **(C)** Expression levels of a prognostic signature comprising of 7 IFN-γGs visualized as a heatmap. **(D)** Survival curves for low and high-risk groups based on OS time. **(E)** The ROC curve for 0.5-, 1-, 2-, 3-, 4-, and 5-year survival. The AUC values of the ROC curves are listed as: 0.704, 0.722, 0.713, 0.746, 0.742 and 0.754.

Using the techniques described in section **Supplementary Figure 3A**, 204 genes exhibiting differential expression were discovered in the C1 and C2 subtypes. Next, we proceeded to conduct a univariate Cox regression analysis on a set of 204 DEGs across subtypes. Following this, we employed LASSO regression and multifactor Cox regression analyses, culminating in the development of a prognostic model comprising seven genes, which included EREG, ADM, IAPP, KRT17, ANXA1, ALB, and C7. The group that underwent training was segmented into two categories, low-risk and high-risk, utilizing suitable cut-off values for risk scores as displayed in **Figure 6A**. Patients with higher risk scores had a greater likelihood of mortality, as evidenced by the survival status and risk score relationship in **Figure 6B**. And **Figure 6C** displays the expression levels of the seven genes included in the prognostic model, as represented in the heatmap. The analysis of patients’ survival indicated that individuals with high-risk scores experienced poorer overall survival (OS), as shown in **Figure 6D**. Additionally, the value for diagnosis of the prognostic model was validated using receiver operating characteristic (ROC) curves, with AUC values of 0.704, 0.722, 0.713, 0.746, 0.742, and 0.754 for 0.5-, 1-, 2-, 3-, 4-, and 5-year survival, respectively, as presented in **Figure 6E**.

### 3.6 Validation performed internally and externally in the prediction model in PC

**Figure 7:**
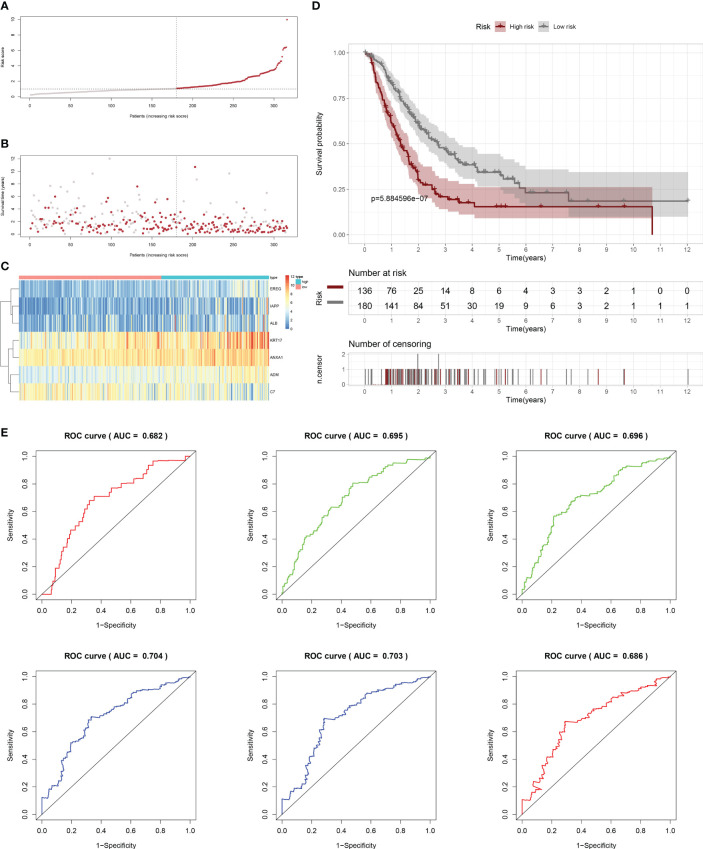
Internal validation results of robust prediction model in Test1 cohort. **(A)** Patients distribution among two risk groups according to appropriate score cut-off values. **(B)** Distribution of survival status and risk scores among patients. (Higher risk scores were associated with increased of mortality). **(C)** Expression levels of a prognostic signature comprising of 7 IFN-γGs visualized as a heatmap. **(D)** Survival curves for low and high-risk groups based on OS time. **(E)** The ROC curve for 0.5-, 1-, 2-, 3-, 4-, and 5-year survival. The AUC values of the ROC curves are listed as: 0.682, 0.695, 0.696, 0.704, 0.703, and 0.686.

**Figure 8:**
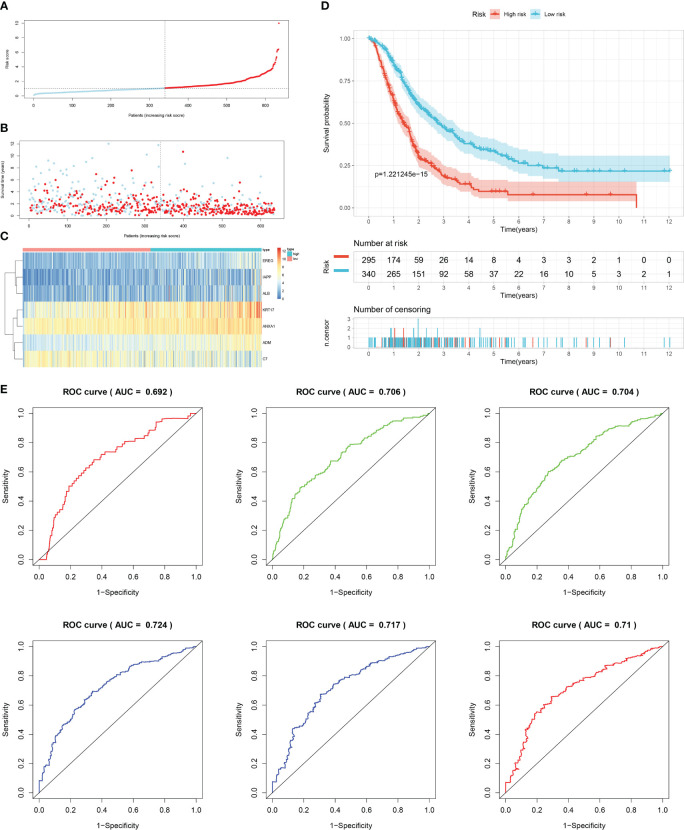
Internal validation results of robust prediction model in Test2 cohort. **(A)** Patients distribution among two risk groups according to appropriate score cut-off values. **(B)** Distribution of survival status and risk scores among patients. (Higher risk scores were associated with increased of mortality). **(C)** Expression levels of a prognostic signature comprising of 7 IFN-γGs visualized as a heatmap. **(D)** Survival curves for low and high-risk groups based on OS time. **(E)** The ROC curve for 0.5-, 1-, 2-, 3-, 4-, and 5-year survival. The AUC values of the ROC curves are listed as: 0.692, 0.706, 0.704, 0.724, 0.717, and 0.710.

**Figure 9:**
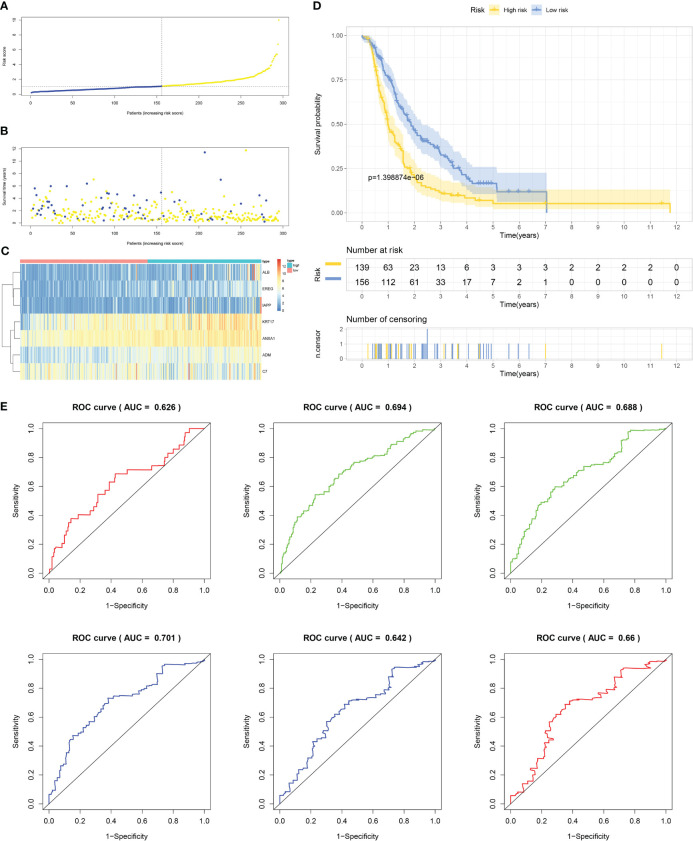
External validation results of robust prediction model in Test3 cohort. **(A)** Patients distribution among two risk groups according to appropriate score cut-off values. **(B)** Distribution of survival status and risk scores among patients. (Higher risk scores were associated with increased of mortality). **(C)** Expression levels of a prognostic signature comprising of 7 IFN-γGs visualized as a heatmap. **(D)** Survival curves for low and high-risk groups based on OS time. **(E)** The ROC curve for 0.5-, 1-, 2-, 3-, 4-, and 5-year survival. The AUC values of the ROC curves are listed as: 0.626, 0.694, 0.688, 0.701, 0.642, and 0.660.

Firstly, patients from test cohorts 1, 2, and 3 were separated into high-risk and low-risk subpopulations using the median risk score in training cohort as the reference. The survival status and risk scores’ distributions in the internal validation cohorts (test 1 and test 2) and external validation cohort (test 3) were similar to those observed in training cohort, as demonstrated in **Figures 7A, B**, **8A, B**, **9A, B**. The heatmaps plotted from the three test cohorts showed that the high-risk group had genes with high expression (EREG, ADM, IAPP, KRT17, ANXA1, and ALB) and genes with low expression (C7) in the internal and external validation cohorts, as shown in **Figures 7C**, **8C**, **9C**. In all three test groups, it was observed that patients with high-risk scores exhibited significantly poorer OS rates, as depicted in **Figures 7D**, **8D**, **9D**. The area under the curve (AUC) values for predicting 0.5-, 1-, 2-, 3-, 4-, and 5-year overall survival were 0.682, 0.695, 0.696, 0.704, 0.703, and 0.686, respectively, in the test 1 cohort, as illustrated in **Figure 7E**. Similarly, in the test 2 cohort, the AUC values were 0.692, 0.706, 0.704, 0.724, 0.717, and 0.710 for the same survival periods, as presented in **Figure 8E**. In the test 3 cohort, the AUC values were 0.626, 0.694, 0.688, 0.701, 0.642, and 0.660 for the same periods, as shown in **Figure 9E**.

### 3.7 Analyzing the immune microenvironment of tumors and drug sensitivity between patients with low and high risk

**Figure 10:**
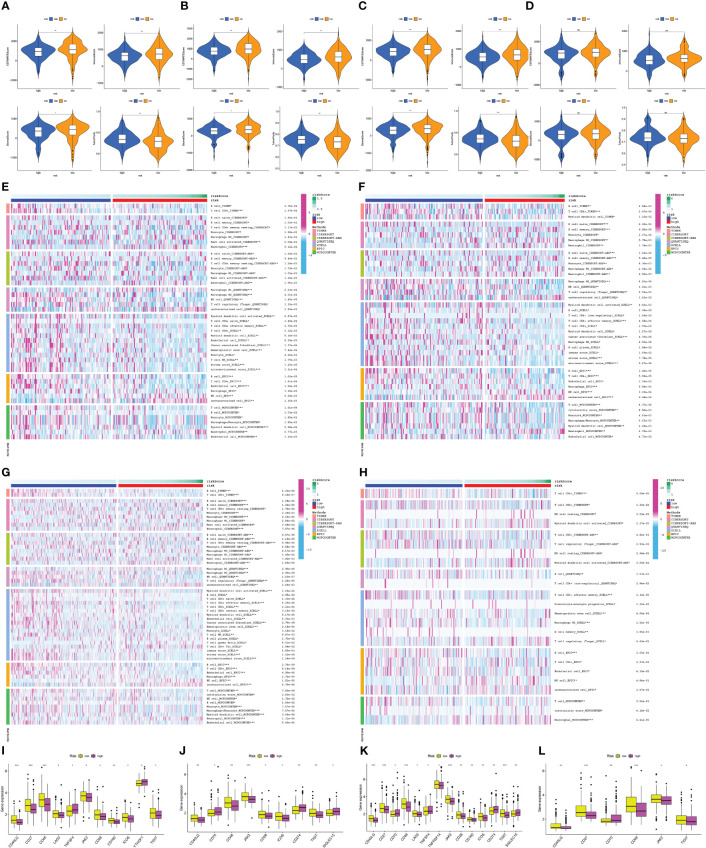
Analysis of tumor immune microenvironment in the high- and low-risk groups for the train, test1, test2, and test3 cohorts. **(A–D)** Comparison of StromalScore, ImmuneScore, ESTIMATEScore, and Tumorpurity between high-risk and low-risk groups in the train, test1, test2, and test3 cohorts. **(E–H)** Comparison of immune cell infiltration levels between low- and high-risk subgroups in different cohorts. Heat maps depict the differences observed in the train, test1, test2, and test3 cohorts. **(I–L)** Box plots comparing immune checkpoint expression in low- and high-risk subgroups of train, test1, test2, and test3 cohorts. (*: p-value < 0.05; **: p-value < 0.01; ***: p-value < 0.001).

We evaluated various scores, namely StromalScore, ImmuneScore, ESTIMATEScore, and Tumorpurity, in both high- and low-risk groups. Our findings unveiled that the low-risk group had greater levels of ImmuneScore, StromalScore, and ESTIMATEScore, while exhibiting lower levels of tumor purity (**Figures 10A–D**). To conduct a more thorough investigation for the immune response, we used various algorithms and generated heatmaps (**Figures 10E–H**). Based on XCELL, stroma score and microenvironment score were higher than that in the low-risk group. Our analysis in the training and test cohorts revealed that low-risk subgroup had higher proportions of anti-tumor immune cells, including B cells, CD4+T cells, CD8+T cells, and M2 macrophages, however, the abundance of immune cell infiltration of neutrophil was upregulated in high-risk group. We also checked the expression of immune checkpoint genes (ICs) in the two groups, as these genes are critical in immunotherapy. Our findings considered that the expression of JAK2, CD48, CD40LG, and TIGIT was downregulated in high-risk group (**Figures 10I–L**).

In the high- and low-risk groups, we carried out a comparison to determine whether there were any variations in chemotherapy sensitivity of patients with regards to the aforementioned targeted therapeutic drugs. Our findings suggest that patients with high-risk PC may experience favorable outcomes from Cisplatin, Docetaxel, Pazopanib, Midostaurin, Epothilone.B, Thapsigargin, Bryostatin.1, and AICAR. Conversely, individuals belonging to the low-risk category in each of the four cohorts. might benefit more from metformin, Roscovitine, Salubrinal, and Cyclopamine (**Supplementary Figure 4**).

### 3.8 Validation of seven model genes via qRT-PCR and HPA platform

**Figure 11:**
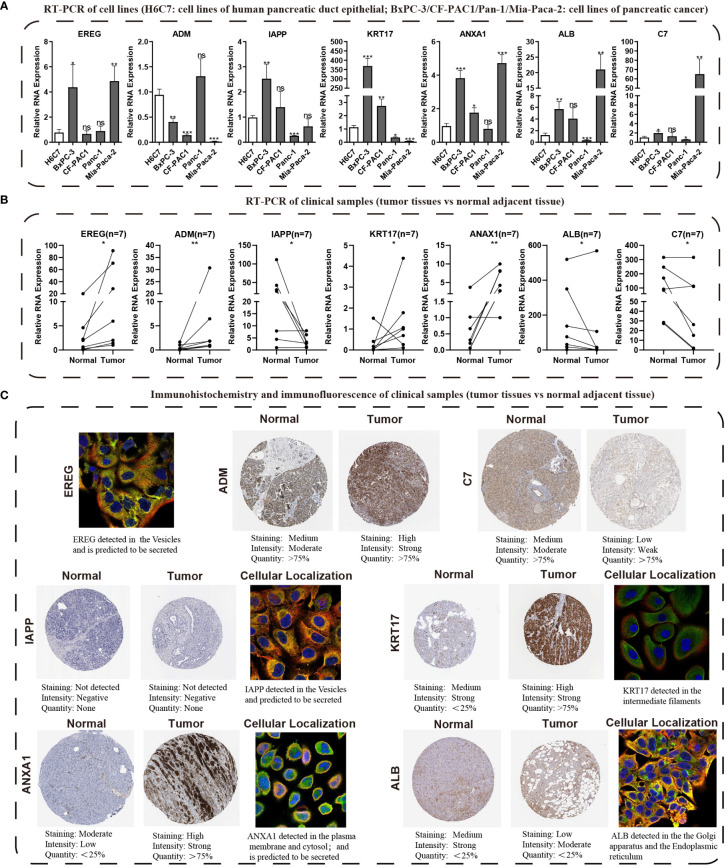
Validating the expression traits of seven central IFN-γGs. **(A)** Analysis of RNA expression levels of EREG, ADM, IAPP, KRT17, ANXA1, ALB and C7 in four PC cell lines (BxPC-3, CF-PAC1, Panc-1, MIA PaCa-2) and H6C7 by the qRT-PCR assays. **(B)** The RNA expression levels of the 7 hub genes in clinical paired samples by the qRT-PCR assays. **(C)** Immunohistochemistry and immunofluorescence of the 7 hub genes from the HPA website(tumor tissues *vs* normal adjacent tissue). (*: p-value < 0.05; **: p-value < 0.01; ***: p-value < 0.001; ns, non-significant).

We conducted KM survival analysis and univariate Cox regression analysis to explore the prognostic significance of the seven model genes furtherly. The results obtained from analyzing the Cox regression univariately indicated EREG, ADM, KRT17, ANXA1, and C7 were significantly associated with PC prognosis, with only C7 being linked to a better prognosis (**Figure S5A**). Notably, KM survival analysis yielded the same outcomes (**Figure S5B**). Heatmaps were then created to demonstrate the expression levels of the model genes in the TCGA, GSE28735, and GSE62452 cohorts (**Figures S5C–E**). qRT-PCR was used to verify the expression patterns of these seven genes in cell lines of PC (**Figure 11A**). Subsequently, we evaluated the expression levels of these seven genes in seven pairs of clinical tissue samples, and the results also revealed differential expression patterns of these genes between cancer and adjacent non-cancerous tissues (**Figure 11B**). The HPA database was utilized to investigate the immunohistochemistry findings of pancreatic tissues, both tumorous and non-tumorous, to assess the protein expression levels of model genes (**Figure 11C**).

## 4 Discussion

PC, a highly fatal malignancy characterized by early metastasis and resistance to anti-cancer therapy, remains a challenge to treat despite improved treatments in recent years, with different subtypes and clinical characteristics affecting prognosis and tumor response (30, 31). Prognostic models could help identify patients who would benefit from more intensive therapies, highlighting the urgent need to identify new molecular biomarkers for diagnosis, prognosis prediction, and treatment response monitoring. Interferons are glycoproteins with antiviral, anti-proliferative, anti-tumor, and immunoregulatory actions, including IFN-γ from the type II IFN family, which has been considered an antitumor candidate because of the function of inhibiting proliferation, inducing apoptosis, and suppressing tumor-derived cytokines by complex mechanisms (32–34). However, clinical trials have not always delivered expected results, and studies conducted in the recent past have revealed that IFN-γ can also contribute to tumor immune evasion, making its role as a therapeutic target for malignancies controversial (35). Further research showed that the tumor IFN-γ induction can both inhibit natural killer (NK) cells by upregulating classical MHC-I molecules and inhibit CD8+ T cells by upregulating non-classical MHC-I molecule Qa-1b. Therefore, it’s valuable to investigate the function of IFN-γ in PC through bioinformatics analysis.

To illustrate the essential function of IFN-γGs in the advancement and growth of human cancers, we conducted a comprehensive analysis of IFN-γGs CNV/SNV variation, expression levels, prognostic factors, methylation levels in human pan-cancer, providing valuable research directions and novel targets for IFN-γGs in the future. Our study placed particular emphasis on PC, investing additional time and effort to conduct a thorough analysis. And we found that some IFN-γGs were actually risk factors for the prognosis of PC, such as TRIM29, OAS1, OAS2 and OASL and so on. On the contrary, some IFN-γGs were actually protective factors for the prognosis of PC, such as NCAM1, TRIM3, PTPN6 and CAMK2B and so on. The conflicting findings could explain the double-edged sword effect of IFN-γ in PC.

We utilized the NMF algorithm to divide 930 PC samples into two clusters based on IFN-γGs. Our results suggested that the C2 subtype with high IFN-γ scores had a significantly better prognosis than C1, suggesting that the IFN-γ pathway may play an anti-cancer role in PC. This conclusion is consistent with previous studies (11, 36, 37), indicating that IFN-γ could inhibit the development of pancreatic cells and may be considered a therapy target for PC patients in the future. And in comparison to the C1 subgroup, the C2 subgroup demonstrates increased expression of IFN-γGs, including HLA-DPB1, HLA-DPA1, HLA-DBQ1, IRF4, IRF8, and JAK2, while exhibiting decreased expression of GBP3. These variations in the expression of IFN-γGs may be a contributing factor to the divergent prognostic outcomes observed in pancreatic cancer patients between the C1 and C2 subgroups. A promising approach for assessing the prognosis and managing individuals with PC involves risk stratification based on the IFN-γ signaling. What’s more, further experiments for the mechanism of these key IFN-γGs need to be carried out.

To explore the potential processes underlying clinical outcome variations among patients with different IFN-γ signaling scores, we thoroughly analyzed the constituent parts of the immune microenvironment and the expression of ICs. TME is a complex combination of tumor, immune, stromal, and extracellular components (38). ImmuneScore, StromalScore, and EstimateScore were analyzed to determine the immune and stromal components of each patient. Our results suggested that the C2 subtype might have a higher immune abundance, which might be a potential reason for its better prognosis. Additionally, after our investigation, we found significant discrepancy in the expression of ICs in the two clusters. We observed significant differences in the expression of ICs between the two subgroups, C1 and C2, such as CD27, CD40LG, CD48, and JAK2, with a notable increase in C2. We believe that these disparities in ICs expression could be one of the reasons contributing to differential outcomes between the two subgroups. Therefore, we conducted further investigations into the relationship between the expression levels of ICs and the prognosis of PC. We found that high expression of immune checkpoints, including CD27, CD40LG, CD48, and JAK2, is indicative of a favorable prognosis for PC. As a result, these ICs that showed differential expression could serve as promising targets for therapeutic intervention. Research has indicated that when immune checkpoint blockers (such as anti-ctLA-4 and/or anti-Pd-1) are used in combination with anticancer vaccines in immunotherapy, it can suppress tumor growth while also increasing the proportion of effector T cells that produce IFN-γ (39). Moreover, PD-1 blockade has been verified to increase the T-cell infiltration by facilitating the IFN-γ-induced chemokines. At the same time, recent research has revealed that IFN-γ could drive the Treg fragility to promote anti-tumor immunity (40). And researchers have found that loss of tumor cell sensitivity to IFN-γ makes tumors more responsive to immune checkpoint blockades (ICBs) in melanoma and pancreatic, lung, renal, and colon cancers (35). Although the anti-tumor effect of IFN-γ in PC is a matter of debate, according to the outcomes of our research and above previous reports, we believe that IFN-γ still plays an anti-tumor role in inhibiting the development of PC and improving patient prognosis. Certainly, we also recognize that the role of IFN-γ in PC is dependent on differences in the TME or other yet-to-be-discovered mechanisms. However, as indicated by the current findings of this study, the IFN-γ or substances that induce IFN-γ production show potential as effective drugs, particularly when used in combination therapy for PC.

Targeted drugs are currently available for various types of tumors, including lung, breast, and ovarian cancers, but they are rarely available for PC (41). Since IFN-γ plays a significant role in PC, we investigated the effectiveness of several commonly used drugs in PC treatment. Our findings indicate that Paclitaxel, Docetaxel, Erlotinib, Midostaurin, Epothilone.B, AICAR, Thapsigargin, and Bryostatin.1 are likely to be beneficial for the C1 subtype, whereas Metformin, Cyclopamine, Roscovitine, and Salubrinal are more beneficial for the C2 subtype.

While molecular typing is essential for functional mining of IFN-γ, it has some shortcomings in type clustering that make it difficult to accurately predict clinical outcomes and IFN-γ scores for individual patients. To address this issue, we utilized LASSO-Cox regression analysis to create a prognostic model comprising of 7 genes that can accurately forecast the overall survival status of PC patients. By applying our model to the training, test1, test2, and test3 cohorts of PC patients, we effectively categorized them into two subgroups based on their risk level and prognosis, with one group having a higher risk and poorer prognosis, while the other group had a lower risk and a better prognosis. Our signature demonstrated strong predictive value, as evidenced by the ROC curves.

Other researchers have also studied the 7 genes and found that they play an important role in PC and other human tumors. EREG, belong to the ERBB family, can stimulate the intrinsic kinase domain of the ERBB1 and ERBB4 receptors, leading to the phosphorylation of certain tyrosine residues in the receptors’ cytoplasmic tail (42). Previous studies have revealed the EREG promotes the proliferation of pancreatic cancer cells and is elevated in cases of PC (43). Patients with PC have been found to have higher serum levels of adrenomedullin, and the ADM gene encodes a peptide hormone that differs between individuals with chronic pancreatitis and healthy ones (44). Knocking down ADM has been shown to reduce myelomonocytic cell recruitment and tumor angiogenesis in pancreatic tumor-bearing mice (45). IAPP, a beta-cell peptide, has been found to have strong anti-tumor effects in p53-deficient tumors by inhibiting glycolysis and proliferation and stimulating apoptosis (46–48). KRT17, a keratin type I family member, has been linked to various malignancies, including PC, and is involved in their occurrence and development (49–54). ANXA1, a calcium-dependent phospholipid-binding protein, playing a significant role in the progress of tumors in various tumor types, including breast cancer, colorectal cancer, and PC (55–59). Low levels of serum ALB, a nutritional status indicator, have been associated with poor prognoses for different cancers (60, 61). The C7 gene produces the 121 kDa serum single-chain glycoprotein C7, which is involved in the membrane assault complex (62). Its role in PC is unclear, but reports have revealed a correlation between C7 expression and poor prognosis in lung tumors and a decrease or even removal of C7 mRNA in esophageal carcinoma cells (63). In this study, we conducted a comparative analysis of mRNA levels for seven prognostic model genes in the H6C7 cell line and four distinct cancer cell lines. Our investigation revealed that, with the exception of ADM, the mRNA expression patterns of the remaining six model genes were consistent with the findings of our bioinformatics analysis in at least one of the pancreatic cancer cell lines. It is important to note that mRNA expression levels of model genes in pancreatic cancer cell lines may deviate from those found in public databases. To address this potential disparity, we procured seven pairs of pathologically confirmed cancer and adjacent tissues from our research center, thereby capturing the mRNA expression profiles of the seven model genes in both cancerous and adjacent tissues. Our observations revealed that ADM and ERRG exhibited significantly elevated total expression levels in human tumor tissues, with expression patterns in all seven pancreatic cancer cases aligning with our predictions. ANXA1 and KRT17 consistently demonstrated expression trends that concurred with our overall bioinformatics analysis in six out of seven pancreatic cancer patients, indicating heightened expression in tumor tissues. Similarly, C7 and ALB displayed expression patterns that were consistent with our overall bioinformatics analysis in six out of seven pancreatic cancer patients, suggesting reduced expression in tumor tissues. Regarding IAPP, in two pancreatic cancer cases, its expression levels in both cancerous and adjacent tissues were nearly identical. However, in the remaining five pancreatic cancer cases, its expression trends mirrored our bioinformatics analysis predictions, indicating differential expression. Considering the inherent variability and heterogeneity in gene expression among individuals, we maintain that our experimental results are accurate and, to a certain extent, validate the credibility and accuracy of our bioinformatics findings. Nevertheless, we acknowledge the necessity of expanding the sample size of pancreatic cancer tissues for further validation. Furthermore, recognizing that proteins constitute the fundamental units of human structural and functional biology, we also assessed the protein expression levels of the model genes. In pancreatic cancer tissues, ADM, KRT17, and ANXA1 protein levels surpassed those in normal samples, while C7 and ALB protein levels exhibited a declining trend, in accordance with the findings from qRT-PCR and bioinformatics analysis. IAPP displayed no significant disparity in protein expression levels between cancerous and adjacent tissues, thus partly corroborating our qRT-PCR results in cells and tissues. However, we acknowledge the need for additional validation with an expanded sample size. In summary, our study provides a comprehensive analysis of the expression patterns of model genes at both the mRNA and protein levels, employing different platforms and examining various pancreatic cancer individuals.

Immunotherapy has emerged as a hopeful approach to treating several types of solid tumors including lung, bladder, and head and neck cancers, which has raised new hope for treating PC. A systematic review has summarized the studies on immunocheckpoint inhibitors (ICIs) in PC (64). The data showed that ICIs in combination with chemotherapy or vaccine therapy could extend the overall survival (OS) of PC to nearly 20 months. Thorough analysis of immune cell infiltration showed that various anti-tumor immune cells, such as B cells and CD4+ T cells, are elevated in the low-risk subgroup, and this aligns with earlier research findings (65, 66). To investigate potential targets for immunotherapy, we analyzed the variations in ICs expression between two groups, as ICs are crucial in immunotherapy. According to our findings, the low-risk group exhibited elevated levels of AK2, CD48, CD40LG, and TIGIT. These ICs are potential therapeutic targets for PC, as they can function as effective inhibitors of immunosuppressive properties. In the future, combination therapy using these ICs inhibitors and agonists may improve the prognosis of PC. Our signature is beneficial for accurately treating people with PC. Patients with high-risk PC may potentially benefit from a combination of Cisplatin, Docetaxel, Pazopanib, Midostaurin, Epothilone.B, Thapsigargin, Bryostatin.1, and AICAR, while those with low-risk PC may benefit from Metformin, Roscovitine, Salubrinal, and Cyclopamine in all subgroups.

Naturally, it is crucial to acknowledge that there are certain constraints to our study. Firstly, our research relied primarily on public databases, which lack clinically relevant information, and therefore the novel prognostic model should be validated with additional real-world prospective data. Secondly, further investigation into the mechanisms of action of the 7 IFN-γGs is required to clarify the extent of their involvement in PC’s development and progression. Thirdly, the significance of IFN-γGs in the TME of PC needs to be deeply explored *in vivo* and *in vitro*. Although our results have limitations, their advantages and clinical significance should not be overlooked. Our research could still offer valuable guidance for basic research and clinical treatment of PC.

## 5 Conclusions

We subdivided PC into two distinct subgroups based on the levels of IFN-γGs expression, thus pioneering a novel prognostic model for PC. Our investigation unveiled that IFN-γGs expression levels exert a partial influence on the TME, drug responsiveness, and the OS of individuals with PC. Certainly, further validation through molecular biology and clinical experiments is imperative to substantiate our discoveries and hypotheses. Nevertheless, our findings concurrently furnish theoretical underpinnings for delving into the crucial mechanisms involving IFN-γGs in PC, and they hold promise in providing personalized guidance for selecting targeted therapies for this malignancy.

## Data availability statement

Upon request, the corresponding authors of this study will provide access to the data used to support the conclusions to relevant parties.

## Ethics statement

The procedures concerning human subjects were carried out in keeping with the ethical guidelines set forth by the relevant research committee and/or institutions, as well as the 1975 Declaration of Helsinki, which was revised in 2013. The Ethics Committee of the First Affiliated Hospital of Dalian Medical University (ID: PJ-KS-KY-2022-60) reviewed and approved the research that included both PC and normal pancreas samples. The studies were conducted in accordance with the local legislation and institutional requirements. The participants provided their written informed consent to participate in this study.

## Author contributions

Each author bears full responsibility for the content and writing of the manuscript. Collectively, the authors made significant contributions to the study’s conception, data gathering and analysis, manuscript writing, and manuscript revision.

## Supplementary material

Supplementary Figure 1 The NMF algorithm utilizes various indicators, including the cophenetic, dispersion, and silhouette measures.

Supplementary Figure 2 The association between the levels of expression of ICs(CD27, CD40LG, CD48, and JAK2) and the prognosis of PC.

Supplementary Figure 3 The detailed process about the DEGs analysis and risk model establishment of PC according to the IFN-γGs. **(A)** The thresholds to identify the DEGs between the C1 and C2(204 DEGs). **(B)** LASSO coefficient profiles of IFN-γGs in PC. **(C)** Model construction’s cross-validation results. **(D)** A total of 7 IFN-γGs are identified by Cox regression analysis.

Supplementary Figure 4 Comparison of chemotherapy sensitivity to targeted therapeutic drugs in the two groups of patients in the train, test1, test2, and test3 cohorts. The drugs could be listed: Cisplatin, Docetaxel, Pazopanib, Midostaurin, Epothilone.B, Thapsigargin, Bryostatin.1, AICAR, metformin, Roscovitine, Salubrinal, and Cyclopamine.

Supplementary Figure 5 The prognostic value and expression level of the 7 model genes. **(A)** Predictive power of the 7 model genes by KM survival analysis. **(B)** Predictive power of the 7 model genes by univariate Cox regression analysis. **(C-E)** The 7 model genes’ expression levels were measured in three cohorts: TCGA, GSE28735, and GSE62452, respectively.
