# Supplementary figures and images for: Identification and characterization of interferon-γ signaling-based personalized heterogeneity and therapeutic strategies in patients with pancreatic cancer
Source: Front Oncol. 2023 Oct 24;13:1227606. doi: 10.3389/fonc.2023.1227606 (PMC10628740; doi:10.3389/fonc.2023.1227606)

# NMF rank survey

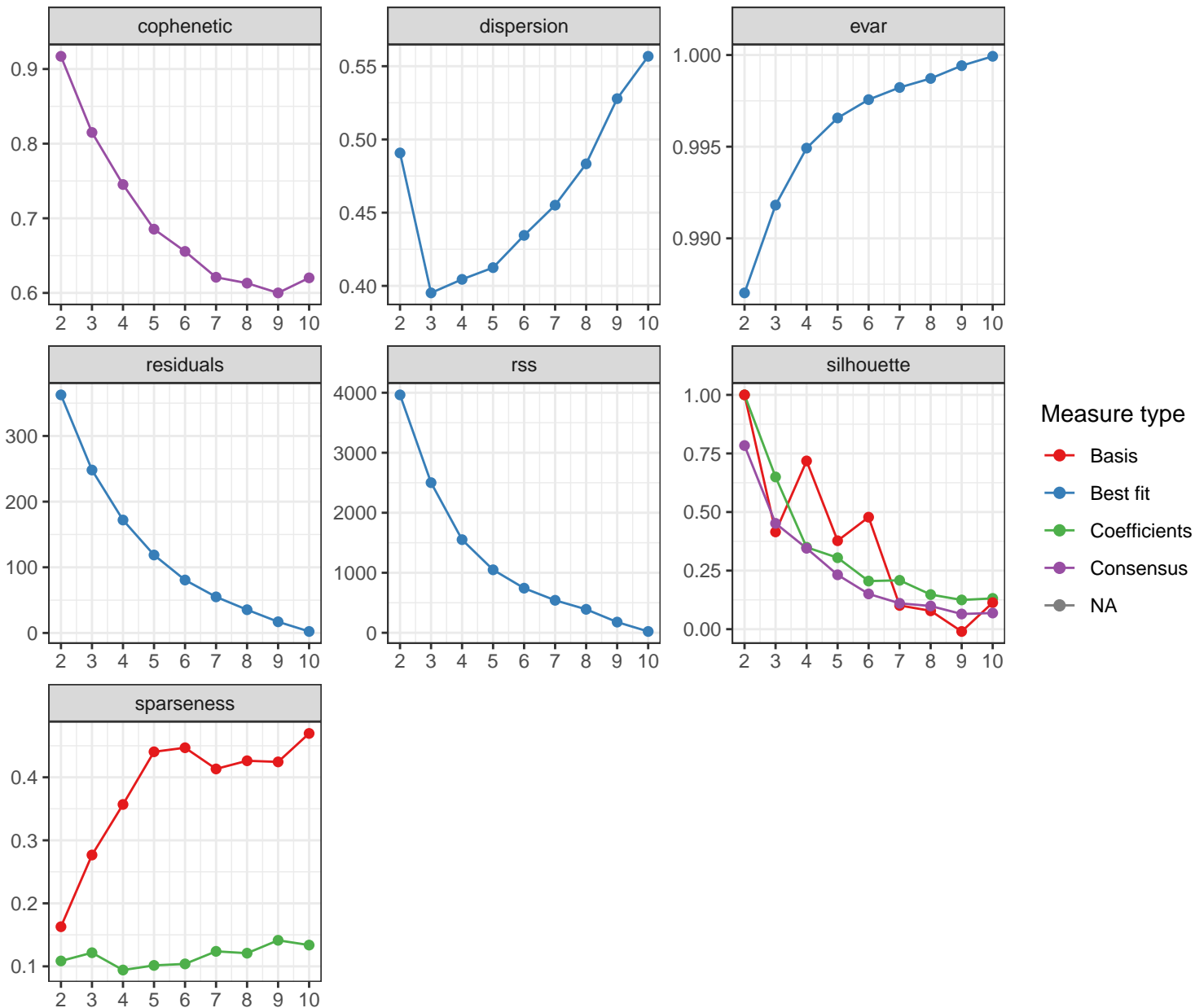

Factorization rank

Supplement: Supplementary Figure 1 — The NMF algorithm utilizes various indicators, including the cophenetic, dispersion, and silhouette measures. [file DataSheet_1.pdf]

CD27 High Low

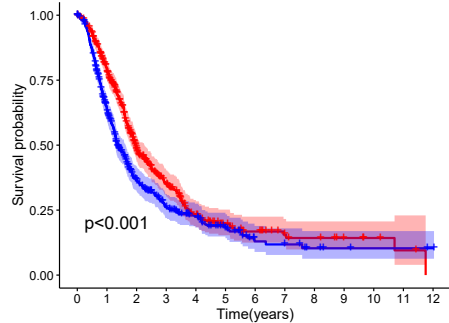

CD40LG High Low

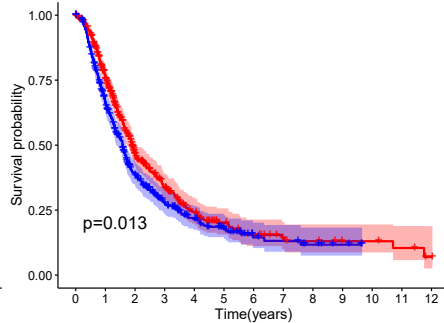

CD48 High Low

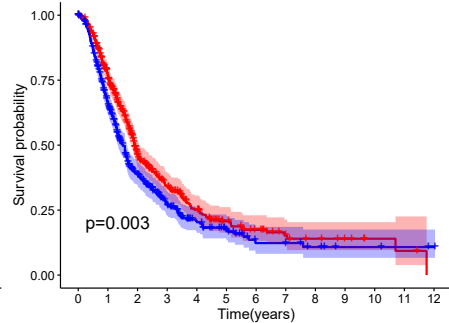

JAK2 High Low

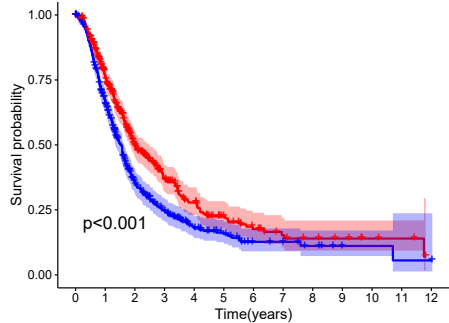

PDCD1LG2 High Low

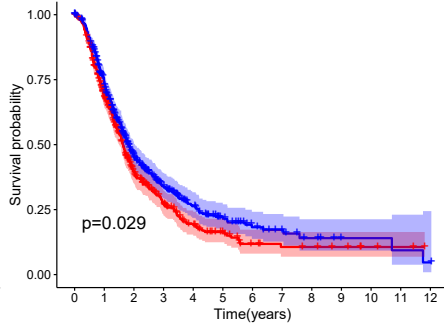

Supplement: Supplementary Figure 2 — The association between the levels of expression of ICs(CD27, CD40LG, CD48, and JAK2) and the prognosis of PC. [file DataSheet_2.pdf]

A

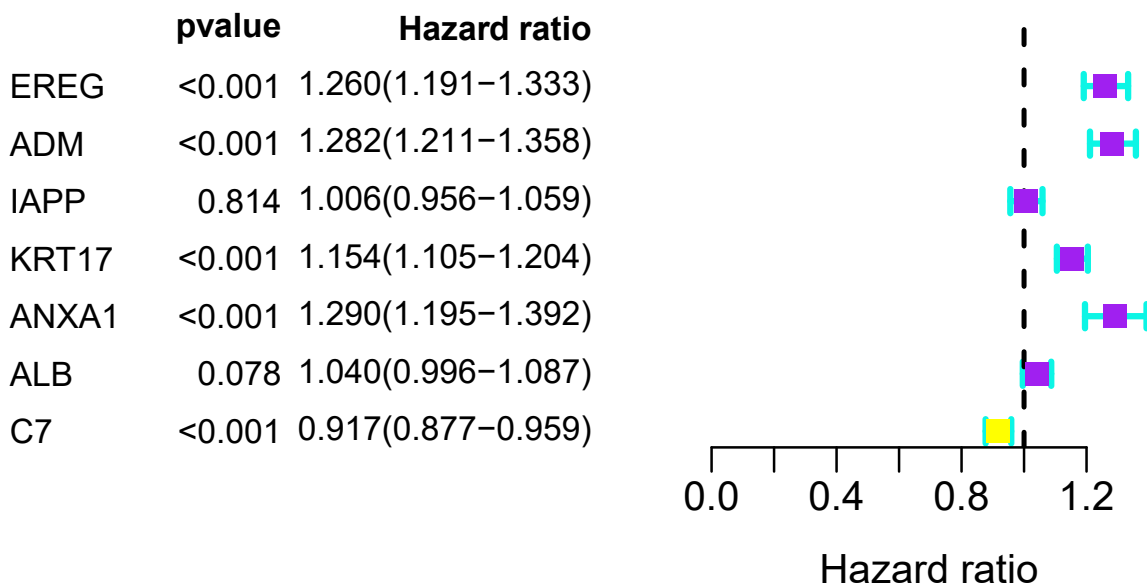

B

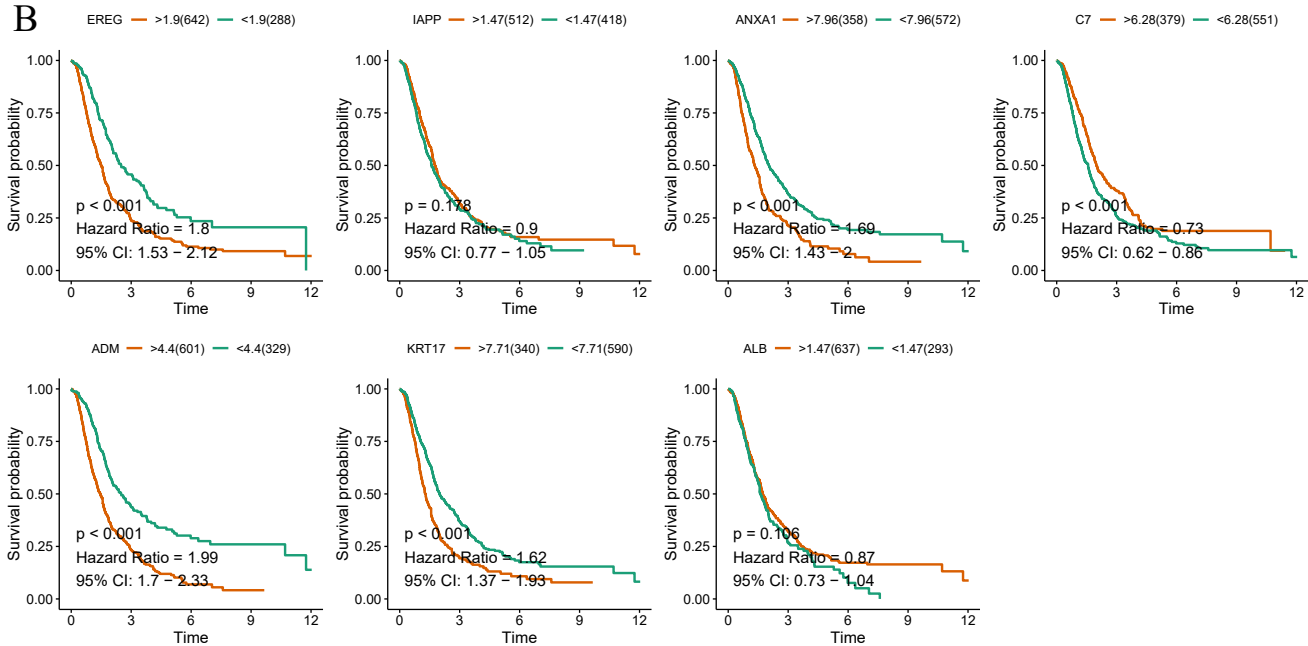

C

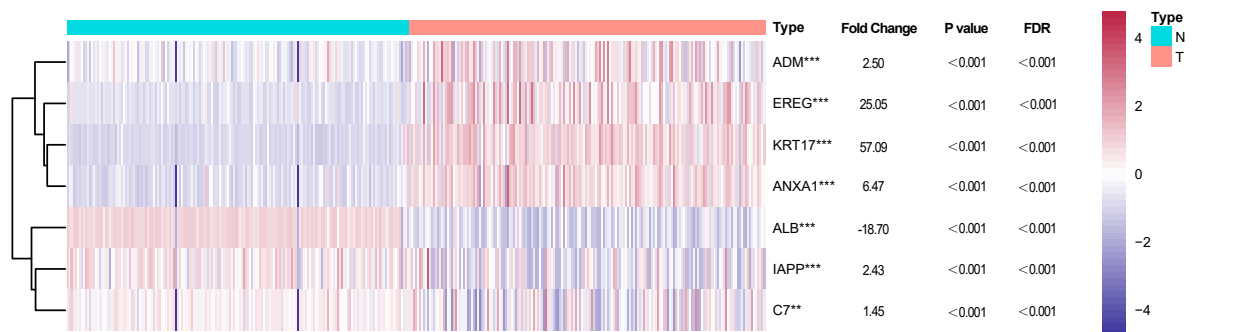

D

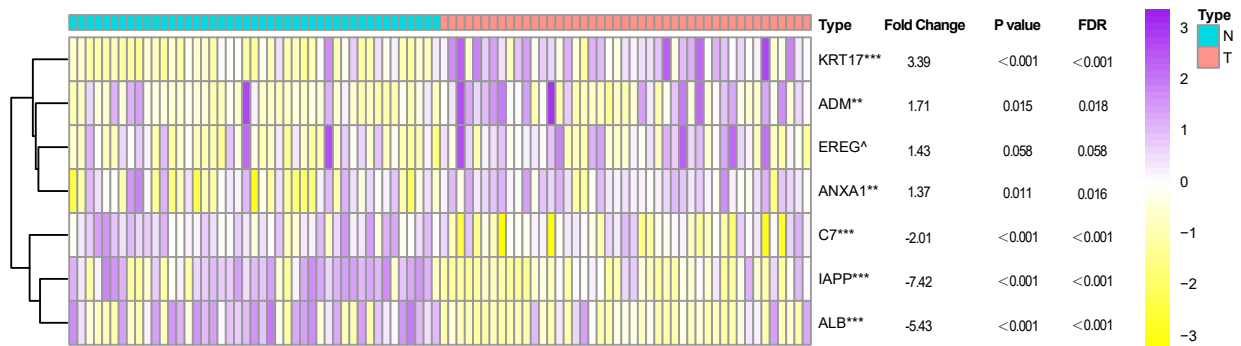

E

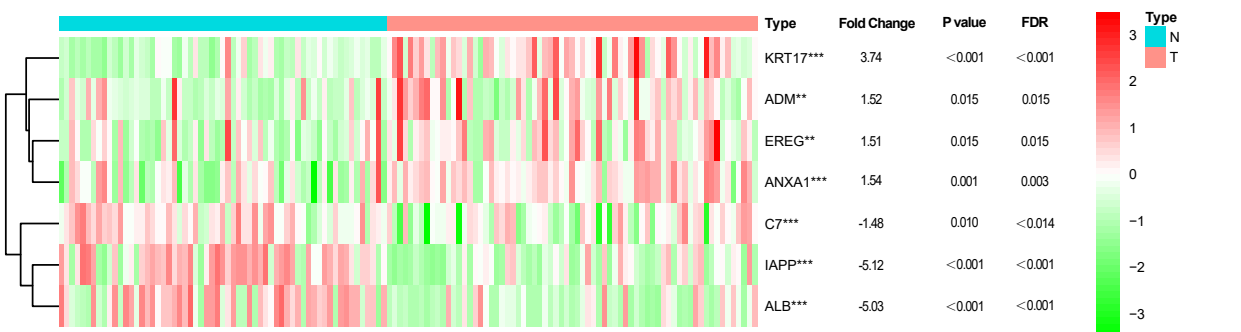

Supplement: Supplementary Figure 5 — The prognostic value and expression level of the 7 model genes. (A) Predictive power of the 7 model genes by KM survival analysis. (B) Predictive power of the 7 model genes by univariate Cox regression analysis. (C-E) The 7 model genes’ expression levels were measured in three cohorts: TCGA, GSE28735, and GSE62452, respectively. [file DataSheet_5.pdf]
