# Supplementary material for: Identification and characterization of interferon-γ signaling-based personalized heterogeneity and therapeutic strategies in patients with pancreatic cancer
Source: Front Oncol. 2023 Oct 24;13:1227606. doi: 10.3389/fonc.2023.1227606 (PMC10628740; doi:10.3389/fonc.2023.1227606)
Supplement: Supplementary Figure 4 — Comparison of chemotherapy sensitivity to targeted therapeutic drugs in the two groups of patients in the train, test1, test2, and test3 cohorts. The drugs could be listed: Cisplatin, Docetaxel, Pazopanib, Midostaurin, Epothilone.B, Thapsigargin, Bryostatin.1, AICAR, metformin, Roscovitine, Salubrinal, and Cyclopamine. [file DataSheet_4.pdf]

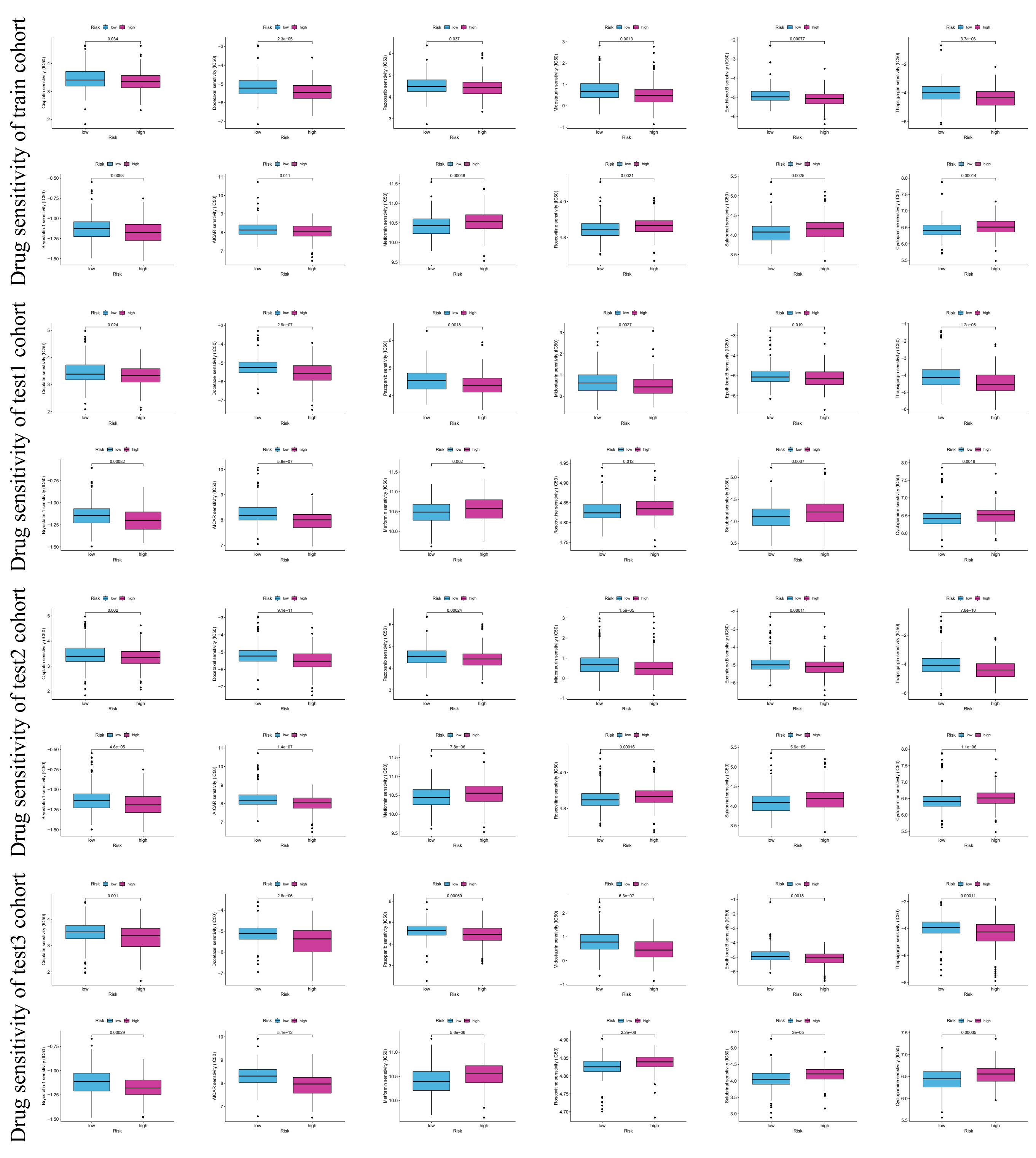
Drug sensitivity of train cohort
Drug sensitivity of test1 cohort
Drug sensitivity of test2 cohort
Drug sensitivity of test3 cohort
Risk low high
Cisplatin sensitivity (IC50)
Docetaxel sensitivity (IC50)
Pazopanib sensitivity (IC50)
Midostaurin sensitivity (IC50)
Epothilone.B sensitivity (IC50)
Thapsigargin sensitivity (IC50)
Bryostatin.1 sensitivity (IC50)
AICAR sensitivity (IC50)
Metformin sensitivity (IC50)
Roscovitine sensitivity (IC50)
Salubrinal sensitivity (IC50)
Cyclopamine sensitivity (IC50)
Risk
low
high
0.034
2.3e-05
0.037
0.0013
0.00077
3.7e-06
0.0093
0.011
0.00048
0.0021
0.0025
0.00014
0.024
2.9e-07
0.0018
0.0027
0.019
1.2e-05
0.00082
5.9e-07
0.002
0.012
0.0037
0.0016
0.002
9.1e-11
0.00024
1.5e-05
0.00011
7.8e-10
4.6e-05
1.4e-07
7.8e-06
0.00016
5.6e-05
1.1e-06
0.001
2.8e-06
0.00059
6.3e-07
0.0018
0.00011
0.00029
5.1e-12
5.6e-06
2.2e-06
3e-05
0.00035
