# Supplementary material for: Identification and characterization of interferon-γ signaling-based personalized heterogeneity and therapeutic strategies in patients with pancreatic cancer
Source: Front Oncol. 2023 Oct 24;13:1227606. doi: 10.3389/fonc.2023.1227606 (PMC10628740; doi:10.3389/fonc.2023.1227606)
Supplement: Supplementary Figure 3 — The detailed process about the DEGs analysis and risk model establishment of PC according to the IFN-γGs. (A) The thresholds to identify the DEGs between the C1 and C2(204 DEGs). (B) LASSO coefficient profiles of IFN-γGs in PC. (C) Model construction’s cross-validation results. (D) A total of 7 IFN-γGs are identified by Cox regression analysis. [file DataSheet_3.pdf]

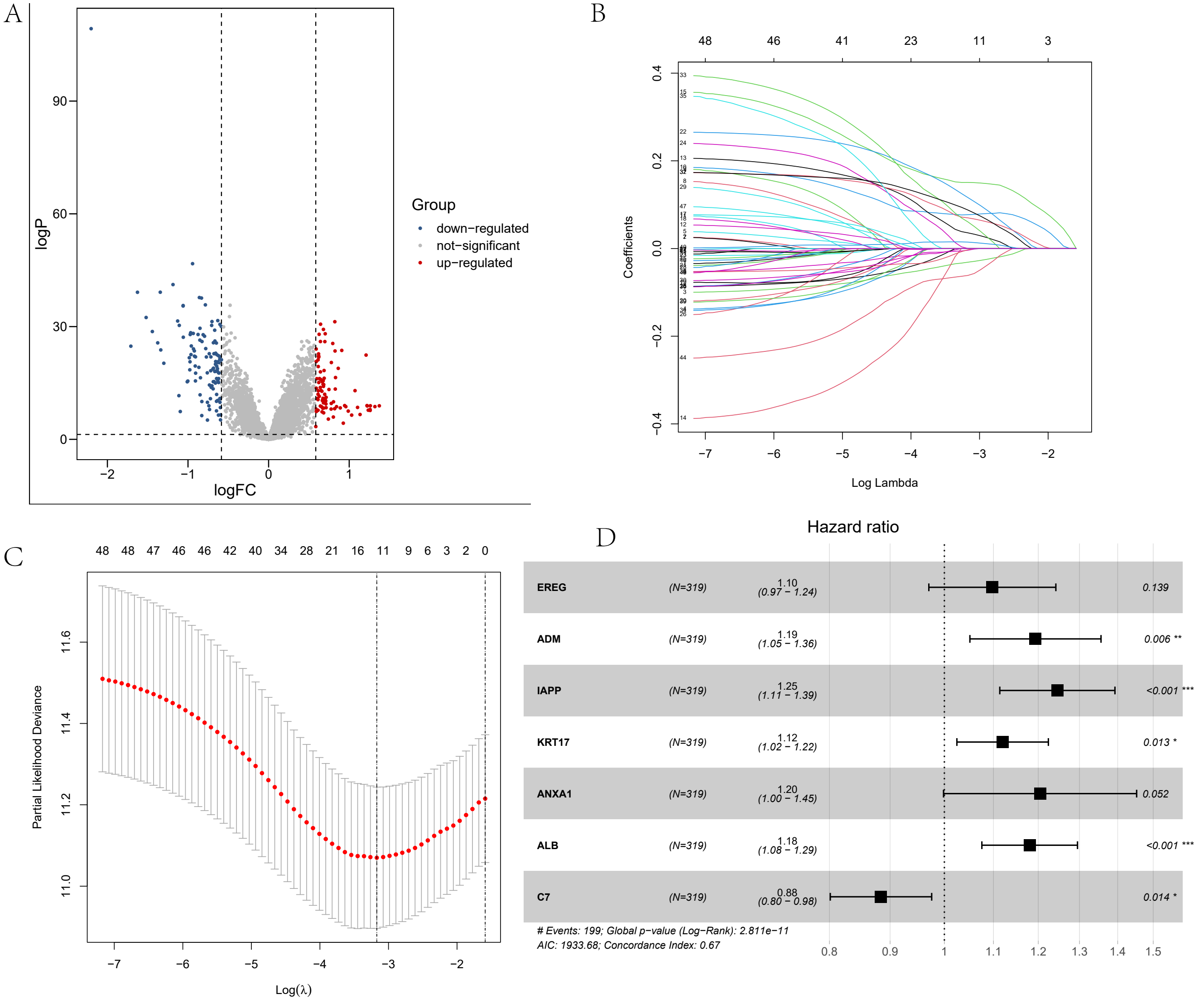
A
logP
logFC
Group
down−regulated
not−significant
up−regulated
B
48 46 41 23 11 3
Coefficients
Log Lambda
C
48 48 47 46 46 42 40 34 28 21 16 11 9 6 3 2 0
Partial Likelihood Deviance
Log(λ)
D
Hazard ratio
EREG (N=319) 1.10 (0.97 − 1.24) 0.139
ADM (N=319) 1.19 (1.05 − 1.36) 0.006 **
IAPP (N=319) 1.25 (1.11 − 1.39) <0.001 ***
KRT17 (N=319) 1.12 (1.02 − 1.22) 0.013 *
ANXA1 (N=319) 1.20 (1.00 − 1.45) 0.052
ALB (N=319) 1.18 (1.08 − 1.29) <0.001 ***
C7 (N=319) 0.88 (0.80 − 0.98) 0.014 *
Events: 199; Global p−value (Log−Rank): 2.811e−11
AIC: 1933.68; Concordance Index: 0.67
